# Molecular evolution of haemagglutinin (*H*) gene in measles virus

**DOI:** 10.1038/srep11648

**Published:** 2015-07-01

**Authors:** Hirokazu Kimura, Mika Saitoh, Miho Kobayashi, Haruyuki Ishii, Takeshi Saraya, Daisuke Kurai, Hiroyuki Tsukagoshi, Komei Shirabe, Atsuyoshi Nishina, Kunihisa Kozawa, Makoto Kuroda, Fumihiko Takeuchi, Tsuyoshi Sekizuka, Hisanori Minakami, Akihide Ryo, Makoto Takeda

**Affiliations:** 1 Infectious Disease Surveillance Center, National Institute of Infectious Diseases, Musashimurayama-shi, Tokyo 208-0011, Japan; 2 Department of Molecular Biodefence Research, Yokohama City University Graduate School of Medicine, Yokohama-shi, Kanagawa 236-0004, Japan; 3 Gunma Prefectural Institute of Public Health and Environmental Sciences, Maebashi-shi, Gunma 371-0052, Japan; 4 Department of Respiratory Medicine, Kyorin University, School of Medicine, Mitaka-shi, Tokyo 181-0004, Japan; 5 Yamaguchi Prefectural Institute of Public Health and Environment, Yamaguchi-shi, Yamaguchi 753-0821, Japan; 6 College of Science and Technology, Nihon University, Chiyoda-ku, Tokyo 101-8308, Japan; 7 Pathogen Genomics Center, National Institute of Infectious Diseases, Musashimurayama-shi, Tokyo 208-0011, Japan.; 8 Department of Obstetrics, Hokkaido University Graduate School of Medicine, Sapporo-shi, Hokkaido 060-8638, Japan; 9 Department of Virology III, National Institute of Infectious Diseases, Musashimurayama-shi, Tokyo 208-0011, Japan

## Abstract

We studied the molecular evolution of the haemagglutinin (*H*) gene (full length) in all genotypes (24 genotypes, 297 strains) of measles virus (MeV). The gene’s evolutionary timescale was estimated by the Bayesian Markov chain Monte Carlo (MCMC) method. We also analysed positive selection sites. The MCMC tree indicated that the MeV *H* gene diverged from the rinderpest virus (same genus) about 250 years ago and that 24 MeV genotypes formed 3 lineages dating back to a 1915 ancestor (95% highest posterior density [HPD] 1882–1941) with relatively rapid evolution (mean rate: 9.02 × 10^−4^ substitutions/site/year). The 3 lineages diverged in 1915 (lineage 1, 95% HPD 1882–1941), 1954 (lineage 2, 95% HPD 1937–1969), and 1940 (lineage 3, 95% HPD 1927–1952). These 24 genotypes may have diverged and emerged between the 1940s and 1990s. Selective pressure analysis identified many negative selection sites on the H protein but only a few positive selection sites, suggesting strongly operated structural and/or functional constraint of changes on the H protein. Based on the molecular evolution of *H* gene, an ancestor MeV of the 24 genotypes emerged about 100 years ago and the structure of H protein has been well conserved.

Measles virus (MeV), which belongs to the genus *Morbillivirus*, the family *Paramyxoviridae*, causes measles, a highly contagious human disease manifesting as high fever, rash, respiratory, and conjunctivitis symptoms^1^. Severe complications may lead to pneumonia and encephalitis^1^.

Due to widespread measles immunisation programmes, the number of measles patients in many countries has been drastically reduced^2^. However, about 145,700 cases were estimated in 2013^2^. The World Health Organization (WHO) is focusing on measles as an eliminative disease, and all six WHO regions have set elimination target years^2^.

To date, 24 MeV genotypes have been confirmed^1^, and the prevalence of each is associated with geographical area^3^. For example, genotype D4 was predominant in European countries from 2010 to 2013, while genotypes D8 and D9 were mainly detected in South-East Asian countries^4^. In China, the great majority of isolates are genotype H1 strains. In addition, genotype B3 is mainly detected in African areas^4^. Thus, although not well understood, the evolution of each MeV genotype differs by area.

The MeV genome encodes two major antigenic proteins, the haemagglutinin (H) and fusion (F) proteins^1^. The H protein regulates viral adsorption and entry, and that of the vaccine strains shows haemagglutination activity in erythrocytes from African green monkeys^1^,^5^. The neutralising antibodies against H protein are protective against MeV infection^6^. Detailed structures of the H protein and the epitopes for the neutralising antibodies have been reported^7^,^8^,^9^,^10^,^11^,^12^,^13^,^14^,^15^,^16^. The amino acid substitutions in H proteins may lead to antigenic changes^10^,^17^ related to the efficacy and development of the vaccine^10^.

Antigenic changes may occur through positive selection pressures in the host^18^. For example, frequent amino acid substitutions resulting from selective immune pressure in the host induce antigenic changes in the attachment glycoprotein (G) of respiratory syncytial virus (RSV)^19^,^20^. These changes are strongly linked to re-infections^21^. On the other hand, our previous report suggested that minor antigenic changes in the H protein are found in some MeV genotypes, such as D3, D5, D9, and H1^22^. However, it is not known if these changes occur in other genotypes of MeV H protein.

Based on these studies, the molecular evolution of the *H* gene of MeV and changes in its antigenicity may be strongly linked. It is essential to be able to predict the antigenic changes of the various MeV genotypes to gain a better understanding of the efficacy of the vaccines. Therefore, we conducted a detailed genetic analysis of the *H* gene and predicted changes in the structure of H protein to gain an understanding of the evolution of the gene in all MeV genotypes.

## Results

### Phylogenetic analysis using the Bayesian Markov chain Monte Carlo (MCMC) and maximum likelihood (ML) methods on the full *H* gene of all measles virus genotypes and rinderpest virus

**Figure 1:**
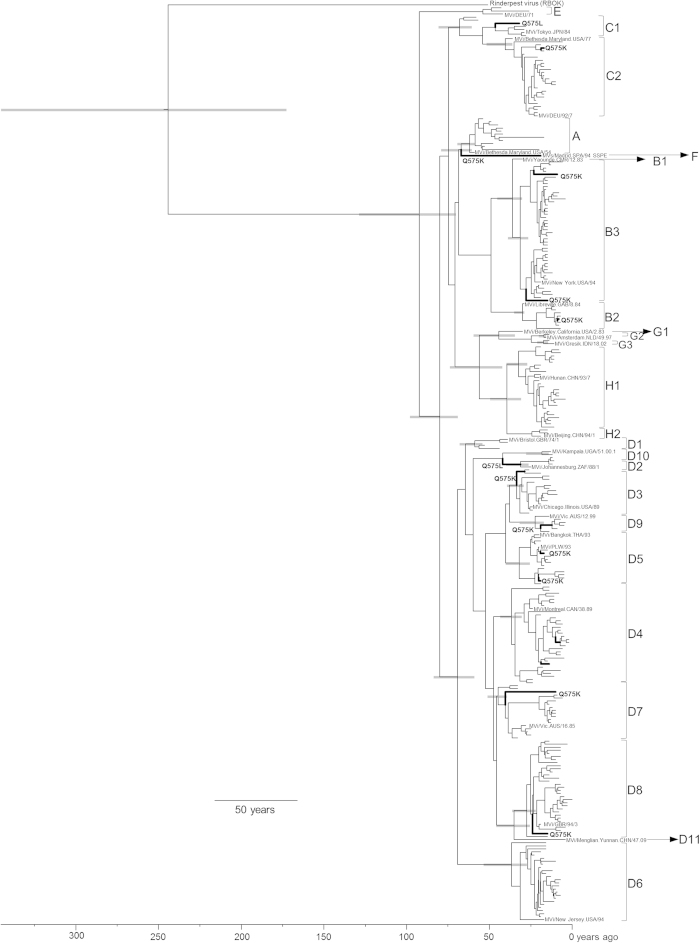
Phylogenetic tree of the haemagglutinin (*H*) gene constructed by the Bayesian Markov chain Monte Carlo method. The Markov chain Monte Carlo tree was based on the full nucleotide sequence of the *H* gene (1854 nt) visualised in FigTree. Grey bars indicate 95% highest posterior density for the estimated year. The tree was estimated using an uncorrelated exponential relaxed clock model under an exponential growth model. The scale bar represents the unit of time (year). The reference strains of each genotype are shown in the tree. Common positively selected sites (aa 575) are indicated by a bold line.

**Figure 2:**
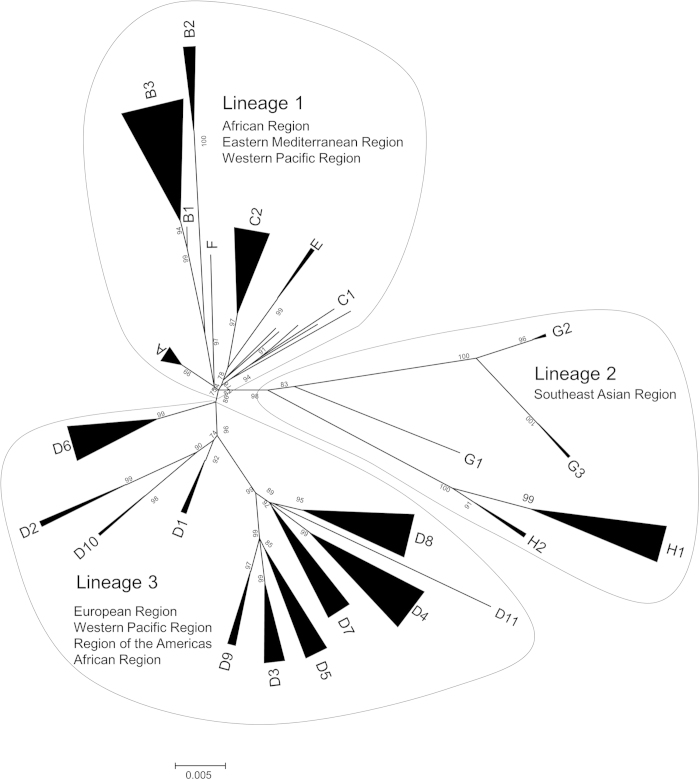
Phylogenetic tree of *H* gene constructed by the maximum likelihood method. Labels at the branch nodes show at least 70% bootstrap support. The scale bar shows nucleotide substitutions per site.

We used 297 strains and constructed the phylogenetic tree using the Bayesian MCMC method with the nucleotide sequences of the *H* gene (1854 nt) for 24 genotypes (A to H) of MeV and a strain of rinderpest virus (Fig. 1). Detailed data of the strains, including lineage, year of divergence, genotype, and rate of evolution, are presented in Table 1. The 95% highest posterior densities (HPDs) for each node of the phylogenetic tree are shown as grey bars in Fig. 1. We estimated the lineages of all genotype strains using a tree constructed by the ML method (Fig. 2). The MCMC tree estimated that the MeV diverged from the rinderpest virus, which belongs to the same genus, at approximately 1760 (95% HPD 1666–1838). These MeV genotype strains formed 3 major lineages (lineages 1 to 3, Fig. 2). Genotypes A, B, C, E, and F were classified as lineage 1. Lineage 2 contains genotypes G and H, and lineage 3 contains genotype D strains (D1–D11). The year of the first major division in the tree was estimated at approximately 1915 (95% HPD 1882–1941). The years of divergence of lineages 1 to 3 were 1915 (95% HPD 1882–1941), 1954 (95% HPD 1937–1969), and 1940 (95% HPD 1927–1952), respectively. All MeV genotypes emerged between the 1940s and 1990s. Details of the years of divergence of each lineage and genotype are shown in Table 1.

Next, the rate of molecular evolution of all genotypes was estimated from the tree as 9.02 × 10^−4^ substitutions/site/year (95% HPD 7.77 × 10^−4^–1.02 × 10^−3^). Divergence for each lineage and genotype was constant and the evolutionary rate was not substantial. The results suggested that MeV has 3 major lineages that uniquely evolved and formed each of the 24 genotypes.

### Positive and negative selection sites in the deduced measles virus H protein

We analysed positive and negative selection sites in the strains using the single likelihood ancestor counting (SLAC), fixed effects likelihood (FEL), and internal fixed effects likelihood (IFEL) methods. The *d*N/*d*S (non-synonymous rate/synonymous rate) values were calculated by these three methods. Detailed data are shown in Tables 2 and 3. First, 6 positive selection sites were found at aa 240, 476, 477, 481, 546, and 575 by the FEL method, 5 positive selection sites were found at aa 240, 282, 303, 315, and 575 by the IFEL method, and 2 positive selection sites were estimated at aa 546 and 575 by the SLAC method. Common sites of positive selection estimated by 3 methods were Q575. The global estimate of *d*N/*d*S was 0.25 (95% confidence interval 0.23–0.27) by the SLAC method. In addition, these common positive selection sites changed at 1940s–2000s (Fig. 1). Many negative selection sites were detected by each of the three methods (Table 3).

**Figure 3:**
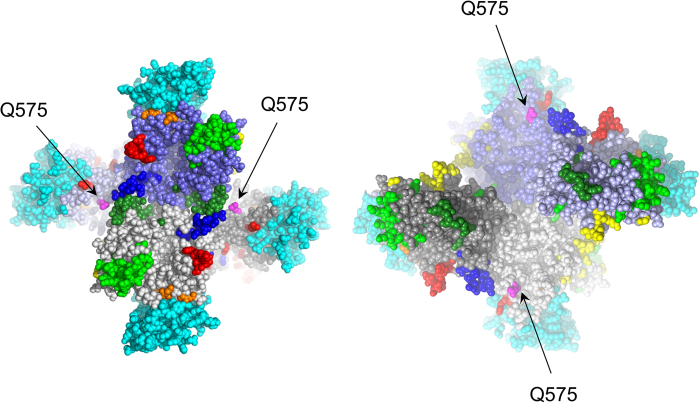
Mapping of the positive selection sites on the H protein structure. An H protein homotetramer determined by Hashiguchi *et al*.^11^ (PDB3ALZ) is shown. (Left) Top view; (Right) bottom view. The individual H proteins in the tetramer are shown in grey, light grey, purple, and light purple, and SLAMs are shown in cyan. Amino acid residues known to constitute a portion of an epitope are shown in blue, green, light green, yellow, orange, and red. The glutamine residues at position 575 (Q575) are shown in magenta.

A structure of a homotetramer (a dimer of dimers) of the H protein complexed with an MV receptor, signalling lymphocyte activation molecule (SLAM)^11^ was shown in Fig. 3. The individual H proteins in the homotetramer were shown in grey, light grey, purple, and light purple, and SLAMs were shown in cyan, as shown in previous papers^14^,^15^. Amino acid residues known to constitute a portion of an epitope were shown in blue, green, light green, yellow, orange, and red, as shown in previous papers^14^,^15^. The glutamine residue at position 575 (Q575) shown in magenta was also located at the surface of the H protein, but was unrelated to known antigenic epitopes (Fig. 3).

### Phylodynamics of measles virus

**Figure 4:**
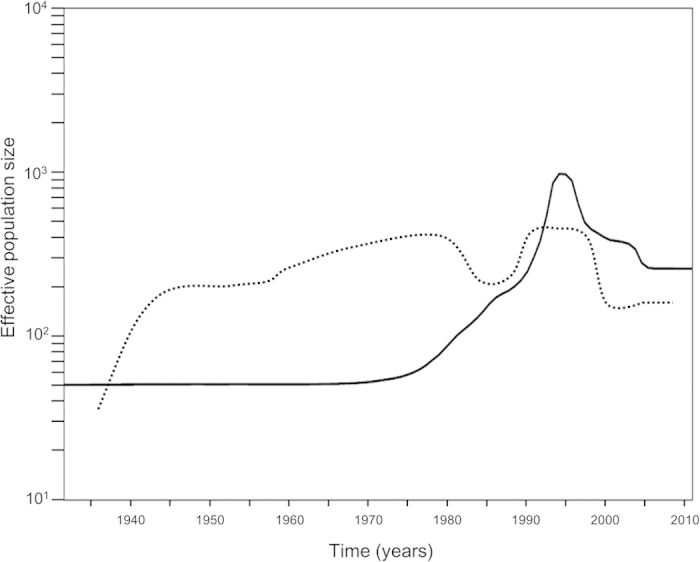
Bayesian skyline plot analysis of the measles virus *H* gene. The Y-axis shows the effective population size and the X-axis shows generation time (year). Mean effective population size of major genotypes including B3, D4, D8, D9, and H1 are indicated by a solid line. Mean effective population size of minor genotypes such as B1, C1, E, and F are indicated by a dotted line.

We assessed the phylodynamics of the *H* gene of several MeV strains using Bayesian skyline plot (BSP) analyses (Fig. 4). Notably, from 1940 to the 1990s, the effective population sizes of the main strains, such as B3, D4, D8, D9, and H1, detected during the past 5 years (major genotypes)^4^ were smaller than those of other minor genotypes. However, the relative population sizes were reversed after 1995. The results suggested that major genotypes more adaptive than minor genotypes during around 15 years.

## Discussion

We studied the molecular evolution of the *H* gene in all known genotypes (24 genotypes, 297 strains) of MeV. First, we found that the MeV diverged from the common ancestors of rinderpest virus about 250 years ago during the 18th century. Twenty-four MeV genotypes were divided into 3 lineages dating back to a 1915 ancestor (95% HPD 1882–1941) based on the time-scaled evolutionary tree obtained using the Bayesian MCMC method. The three lineages diverged in 1915 (lineage 1, 95% HPD 1882–1941), 1954 (lineage 2, 95% HPD 1937–1969), and 1940 (lineage 3, 95% HPD 1927–1952). The 24 genotypes might have diverged and emerged between the 1940s and 1990s. Only a small number of positive selection sites were found, although many negative selection sites were identified. In addition, the recently effective population sizes of major genotypes (B3, D4, D8, D9, and H1)^4^ detected during the past 5 years were larger than those of minor genotypes. From the molecular evolution of the *H* gene, an ancestor MeV of these 24 genotypes emerged about 100 years ago, and the H protein structure has been well conserved. Such H protein conservation must be partially responsible for the effectiveness of the present vaccine.

Phylogenetic analysis in this study clearly showed the 3 lineages (Fig. 2). The strains belonging to lineage 1 form eight genotypes and are mainly detected in African, eastern Mediterranean, and western Pacific areas, while the strains of lineage 2 form five genotypes and are detected in Southeast Asian areas^4^. In contrast, the strains belonging to lineage 3 are detected across the world, including European, African, Asian, and American areas^4^. The strains of lineage 3 form many genotypes (D1 to D11), and genotypes D4, D8, and D9 are detected in many countries^4^. We also estimated the year of divergence of each MeV genotype, including rinderpest virus, using the Bayesian MCMC method. As a result, we found that MeV *H* gene diverged from a common ancestor of the rinderpest virus about 250 years ago (Fig. 1). After that, MeV uniquely evolved and formed 24 genotypes. Genotype C1 putatively emerged in 1942, the earliest year of divergence, while genotype G2 was the latest to emerge in 1994. In addition, Furuse *et al*., estimated that MeV diverged from rinderpest virus at around 11th to 12th centuries based on *N* (nucleoprotein) gene analysis using MCMC method^23^. Their and our results suggested that the genetic histories of the *H* and *N* genes might be distinct, although analysis of other genes may be needed to more fully describe the MeV genome.

Some prevalent strains, B3, D4, D8, D9, and H1, putatively emerged during the 1970s to 1980s. However, these genotypes have recently been detected as major genotypes. In contrast, some genotypes such as B1, E, F, G1, and G2 have rarely been detected the last 10 years^4^,^24^. These major genotypes showed wide genetic divergence in phylogenetic trees. In addition, BSP analysis suggested that the effective population sizes of the major genotypes were larger during the most recent 5 years than those of the minor genotypes (Fig. 4). These results suggest that the ancestor of these major genotypes might be more adaptive to humans, although we analysed only the *H* gene.

Previous reports have discussed the evolution rates of the *H* gene in MeV as ranging from 7.28 × 10^−6^ to 6 × 10^−3^ substitutions/site/year^22^,^25^,^26^,^27^. In the present study, we estimated that the mean rate of the molecular evolution of MeV *H* gene as 9.02 × 10^−4^ substitutions/site/year. While the present data were obtained from all 24 genotypes, other data were based on limited genotypes of MeV^22^,^25^,^26^,^27^. Thus, the variation among data from these studies may be partially attributed to the genotypes analysed.

We estimated positive and negative selection sites in the gene by SLAC, FEL, and IFEL methods. Positive selection imparts a survival advantage under the selective constraints that confront the viral population^18^. Negative selection plays an important role in maintaining the long-term stability of biological structures by removing deleterious mutations^18^,^28^. In this study, some positive selection sites were found (Table 2). A previous report showed that there are 14 positive selection sites in 50 strains of the *H* gene in various genotypes^29^. In addition, our previous report showed that the prevalent Asian genotypes, D3, D5, D9, and H1, have 8 positive selection sites estimated by FEL or IFEL methods^22^. Of these findings, amino acid positions corresponding to aa 476 and aa 575 are compatible with our results. In the previous report, aa 463–477 involving aa 476, and aa 561–575 involving aa 575, react with human sera for the neutralisation of MeV H protein^22^. Furthermore, aa 463–477 is a possible candidate for the binding site of the CD46 receptor^22^. The amino acid change at positive selection site 575 may confer an advantage to MeV transmission. However, the residue at position 575 was unrelated to the known effective neutralising epitopes. Therefore, an amino acid substitution at this position may not substantially affect the efficacy of humoral immunity against MeV. Nevertheless, only a small number of positive selection sites were identified for the MeV H protein, and there is little evidence to show that amino acid substitutions on the epitopes impart substantially better fitness to MeV *in vivo*. These observations could provide a rationale for the high level of efficacy of currently used MeV vaccines against all circulating MeV strains.

Many negative selection sites were found among the strains (Table 3). In general, negative selection is responsible for the prevention of functional deterioration of the viral proteins^18^. For example, negative selection sites in the neutralisation epitopes of polioviruses may be involved in receptor recognition and in the formation of altered particles^30^. Although the roles of these sites in MeV H protein are not exactly known, it is possible that these substitutions are involved in preventing the functional deterioration of major antigen (H protein)^14^,^15^.

In conclusion, the results suggest that the 24 MeV genotypes were generated over approximately 90 years and that the H protein was well conserved. This protein is an essential antigen of MeV. Thus, analysis of the biological properties of MeV H protein is required through the virus surveillance system. These studies will allow us to estimate the molecular evolution of MeV and give a better understanding of the aetiology of MeV.

## Materials and methods

### Strains and alignments

All collected sequence data were available in the DDBJ/GenBank database. We gathered a comprehensive collection of the full-length coding sequences for *H* gene of MeV. Sequence alignment was performed by CLUSTAL W^31^. Strains with 100% identity were excluded from the dataset. Furthermore, rinderpest virus (RBOK strain, Genbank accession No. Z30697) was added to the dataset. Accordingly, 297 strains of MeV and 1 strain of rinderpest virus were used in this study. The nucleotide sequences corresponded to positions 1–1854 of the *H* gene in strain MVi/Bethesda.Maryland.USA/54 (GenBank accession No. M81895). The detailed data are shown in Supplementary Table S1.

### Phylogenetic analysis using the Bayesian Markov chain Monte Carlo method

To analyse the time-scaled phylogeny and evolutionary rate of *H* gene, we used the MCMC method in BEAST v1.7.5^32^. The best nucleotide substitution model was selected using KAKUSAN4^33^. The clock model and demographic model were compared by calculating Akaike’s information criterion through MCMC (AICM)^34^,^35^, for each model using Tracer v1.6^36^. In this study, we tested 4 clock models: a strict clock, an uncorrelated lognormal relaxed clock, an uncorrelated exponential relaxed clock, and a random local clock^37^. Two demographic models, constant size model and exponential growth model, were also compared using AICM calculation^34^,^35^. We employed the model with the lowest AICM value. As a result, the datasets were analysed using a GTR-Γ model under an uncorrelated exponential relaxed clock with an exponential growth model. The data of each model are shown in Table S2. The MCMC chains were run for 75,000,000 steps and sampled every 10,000 steps. Convergence was assessed from the effective sample size (ESS) after a 10% burn-in using Tracer v1.6^36^. ESS values above 200 were accepted. Three BEAST runs were combined using LogCombiner (available in BEAST package) following a 10% burn-in from each file. Uncertainty in the estimates was indicated by the 95% HPD intervals. The maximum clade credibility tree was generated by Tree Annotator v 1.7.4 after a 10% burn-in. The phylogenetic tree was viewed in FigTree v1.3.1^29^. The evolutionary rates of each lineage were also estimated as described.

### Phylogenetic analysis by the maximum likelihood methods

The phylogenetic tree of all MeV genotypes was constructed using the ML method with PhyML3.0^38^. The best substitution model was selected using KAKUSAN4^33^, and we employed the GTR-Γ model. The reliability of the tree was estimated using 1000 bootstrap replications.

### Selective pressure analysis

Selection pressure analysis of the *H* gene was performed using Datamonkey^39^. We calculated the *d*S and *d*N rates at every codon using the following 3 methods: SLAC, FEL, and IFEL. The SLAC and FEL methods detect sites under selection at external branches of the phylogenetic tree, while the IFEL method investigates sites along the internal branches. SLAC is more conservative than other methods and intensive for large alignments^40^. However, this method appears to underestimate the substitution rate^40^. In contrast, FEL and IFEL methods consider both synonymous and nonsynonymous rate variations and may be efficiently parallelized^40^. Thus, we used the 3 different methods to obtain an accurate estimate of positive selection sites in the present study^19^,^41^. Positive (*d*N > *d*S) and negative (*d*N < *d*S) selection sites were determined using a *p*-value of <0.05.

### Mapping of positive selection sites on the H protein structure

To clarify the positions of substituted amino acids at the positive selected sites on the H protein, we mapped them on the H protein structure reported by Hashiguchi *et al*.^11^. Figures were produced using PyMOL (DeLano Scientific LLC, Palo Alto, CA, USA. http://www.pymol.org).

### Bayesian Skyline Plot analysis

To assess the differences in phylodynamics among each genotype, a BSP was constructed using the BEAST program^32^,^42^. We compared the effective population sizes of prevalent types^4^,^24^ such as B3, D4, D8, D9, and H1 (major genotypes, 159 strains) with those of other minor genotypes (147 strains). A substitution model was selected as described above. The MCMC chains were run for 150,000,000 steps with sampling every 10,000 steps under the strict clock, GTR-Γ model.

## Additional Information

**How to cite this article**: Kimura, H. *et al*. Molecular evolution of haemagglutinin (*H*) gene in measles virus. *Sci. Rep*. **5**, 11648; doi: 10.1038/srep11648 (2015).

## Supplementary Material

Supplementary Information

## Figures and Tables

**Table 1:** Year of divergence of each genotype.

	Genotypes	Year of divergence (95% HPD)
1	A, B1, B2, B3, C1, C2, E, F	1915 (1882–1941)
2	G1, G2, G3, H1, H2	1954 (1937–1969)
3	D1, D2, D3, D4, D5, D6, D7, D8, D9, D10, D11	1940 (1927–1952)
A		1948 (1941–1952)
B1		1974 (1966–1980)
B2		1980 (1976–1982)
B3		1979 (1972–1984)
C1		1942 (1930–1950)
C2		1969 (1959–1975)
D1		1951 (1943–1957)
D2		1979 (1971–1985)
D3		1977 (1972–1982)
D4		1974 (1968–1980)
D5		1978 (1971–1985)
D6		1973 (1957–1984)
D7		1966 (1960–1971)
D8		1983 (1975–1989)
D9		1988 (1979–1994)
D10		1992 (1983–1997)
D11		1976 (1965–1986)
E		1955 (1937–1965)
F		1943 (1932–1950)
G1		1965 (1951–1976)
G2		1994 (1991–1995)
G3		1993 (1990–1996)
H1		1978 (1971–1984)
H2		1986 (1977–1991)

HPD, highest posterior density.

**Table 2:**
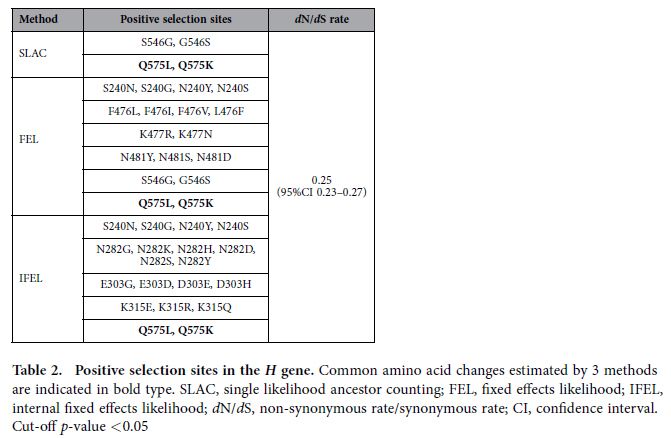
Positive selection sites in the *H* gene.

**Table 3:** Negative selection sites in the *H* gene.

Method	Negative selection sites
SLAC	181 sites
FEL	242 sites
IFEL	157 sites

Cut-off *p*-value <0.05
