# Supplementary material for: Molecular evolution of haemagglutinin (H) gene in measles virus
Source: Sci Rep. 2015 Jul 1;5:11648. doi: 10.1038/srep11648 (PMC4486977; doi:10.1038/srep11648)
Supplement: Supplementary Information [file srep11648-s1.pdf]

Molecular evolution of haemagglutinin (*H*) gene in measles virus

Hirokazu Kimura*, Mika Saitoh, Miho Kobayashi, Haruyuki Ishii, Takeshi Saraya, Daisuke Kurai, Hiroyuki Tsukagoshi, Komei Shirabe, Atsuyoshi Nishina, Kunihisa Kozawa, Makoto Kuroda, Fumihiko Takeuchi, Tsuyoshi Sekizuka, Hisanori Minakami, Akihide Ryo, and Makoto Takeda*

*Corresponding authors

Dr. Hirokazu Kimura, Infectious Disease Surveillance Center, National Institute of Infectious Diseases, 4-7-1 Gakuen, Musashimurayama-shi, Tokyo 208-0011, Japan

Phone: +81-42-561-0771; Fax: +81-42-565-3315; E-mail: kimhiro@nih.go.jp

Dr. Makoto Takeda, Department of Virology III, National Institute of Infectious Diseases, 4-7-1 Gakuen, Musashimurayama-shi, Tokyo 208-0011, Japan

Phone: +81-42-561-0771; Fax: +81-42-565-3315; E-mail: mtakeda@nih.go.jp

Fig. S1. Comparison of Bayesian skyline plot analyses of major genotypes and minor genotypes of the measles virus *H* gene.

(Left) Minor genotypes; (Right) major genotypes. The solid black lines represent mean effective population sizes. The dotted lines show the 95% highest posterior density intervals.

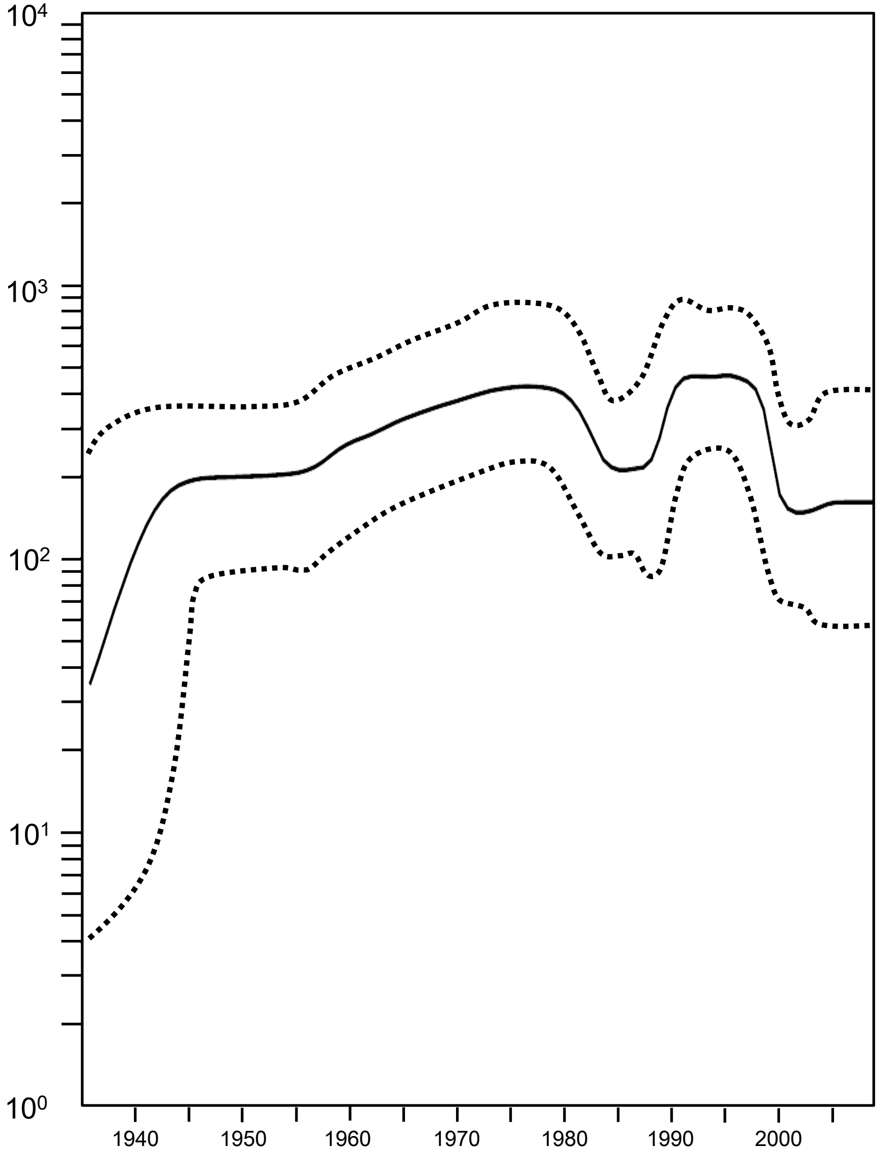

10^4
10^3
10^2
10^1
10^0
1940
1950
1960
1970
1980
1990
2000

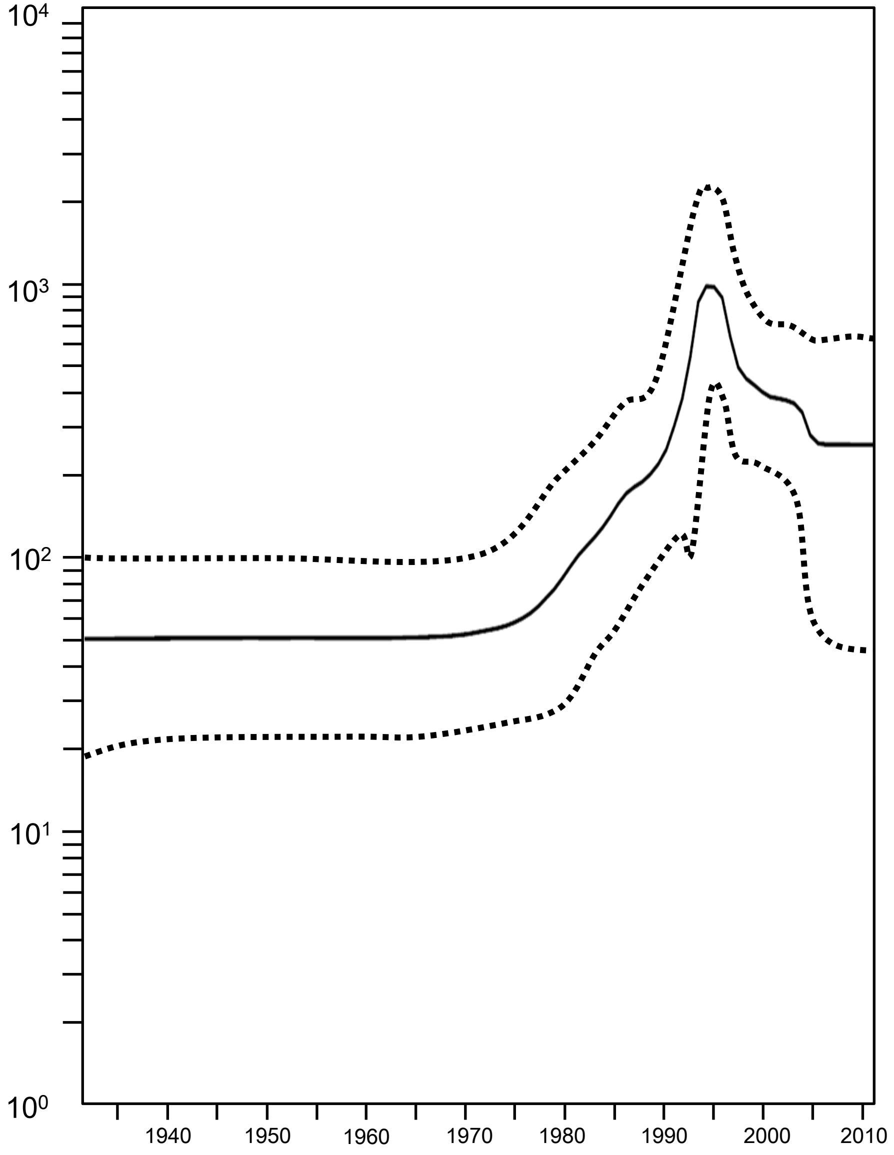

10^4
10^3
10^2
10^1
10^0
1940
1950
1960
1970
1980
1990
2000
2010

Table S1. MeV strains used in this study

Genotype	Strain	Country	Collection year	Genbank accession No.
A	MVi/Bethesda.Maryland.USA/54	United States	1954	U03669
A	MVi/USA/64	United States	1964	Z80791
A	MVi/Pennsylvania.USA/70/3	United States	1970	U08417
A	MVi/USA/71	United States	1971	L46870
A	MVi/USA/71/2	United States	1971	X04720
A	MVi/Delaware.USA/8.96	United States	1996	AF009600
A	MVi/Changchun.Jilin.CHN/65	China	1965	U03652
A	MVi/Shandong.CHN/93/5	China	1993	AF045204
A	MVi/IRL/71	Ireland	1971	Z80790
A	MVi/Osaka.JPN/59	Japan	1959	AB045396
A	MVi/JPN/68	Japan	1968	U03649
A	MVi/RUS/60	Russia	1960	U03660
B1	MVi/Yaounde.CMR/12.83	Cameroon	1983	AF079552
B2	MVs/Kinshasa.COD/42.05/3	Congo	2005	HM802035
B2	MVi/Kinshasa.COD/42.05/4	Congo	2005	HM802036
B2	MVi/Libreville.GAB/8.84	Gabon	1984	AF079551
B2	MVs/Cape Town.SOA/45.02	South Africa	2002	AY994535
B2	MVi/Mbujimayi.COD/06.06/4	Congo	2006	HM802026
B2	MVi/Mbujimayi.COD/06.06/2	Congo	2006	HM802020
B2	MVs/Bas-Congo.COD/06.06/5	Congo	2006	HM802019
B2	MVs/Bas-Congo.COD/06.06/9	Congo	2006	HM802021
B2	MVs/Luanda.AGO/13.03/1	Angola	2003	AY994537
B3	MVi/New York.USA/94	United States	1994	L46752
B3	MVi/New Jersey.USA/45.05	United States	2005	JN635408
B3	MVi/Ibadan.NGA/97/1	Nigeria	1997	AJ239133
B3	MVi/Lagos.NGA/97	Nigeria	1997	AJ239173
B3	MVi/Ibadan.NGA/97/3	Nigeria	1997	AJ239134
B3	MVi/Ibadan.NGA/10.98/4	Nigeria	1998	AJ239165
B3	MVi/Ibadan.NGA/10.98/5	Nigeria	1998	AJ239166
B3	MVi/Ibadan.NGA/10.98/6	Nigeria	1998	AJ239167
B3	MVi/Ibadan.NGA/7.98/1	Nigeria	1998	AJ239135
B3	MVi/Ibadan.NGA/7.98/2	Nigeria	1998	AJ239136
B3	MVi/Ibadan.NGA/7.98/3	Nigeria	1998	AJ239137
B3	MVi/Ibadan.NGA/8.98/10	Nigeria	1998	AJ239146
B3	MVi/Ibadan.NGA/8.98/11	Nigeria	1998	AJ239147
B3	MVi/Ibadan.NGA/8.98/2	Nigeria	1998	AJ239139
B3	MVi/Ibadan.NGA/8.98/8	Nigeria	1998	AJ239144
B3	MVi/Ibadan.NGA/8.98/1	Nigeria	1998	AJ239138
B3	MVi/Ibadan.NGA/9.98/12	Nigeria	1998	AJ239160
B3	MVi/Ibadan.NGA/9.98/3	Nigeria	1998	AJ239151
B3	MVi/Ibadan.NGA/9.98/4	Nigeria	1998	AJ239152
B3	MVi/Ibadan.NGA/9.98/5	Nigeria	1998	AJ239153
B3	MVi/Ibadan.NGA/9.98/6	Nigeria	1998	AJ239154
B3	MVi/Ibadan.NGA/9.98/7	Nigeria	1998	AJ239155
B3	MVi/Ibadan.NGA/9.98/8	Nigeria	1998	AJ239156
B3	MVi/Lagos.NGA/10.98	Nigeria	1998	AJ239170
B3	MVi/Lagos.NGA/11.98/1	Nigeria	1998	AJ239171
B3	MVi/Lagos.NGA/8.98	Nigeria	1998	AJ239169
B3	MVi/Ibadan.NGA/8.98/7	Nigeria	1998	AJ239143
B3	MVi/Accra.GHA/98	Ghana	1998	AJ239177
B3	MVi/Accra.GHA/9.98/1	Ghana	1998	AJ239174
B3	MVi/Accra.GHA/9.98/2	Ghana	1998	AJ239175
B3	MVi/Almeria.SPA/11.03/3	Spain	2003	FJ865561
B3	MVi/Lyon.FRA/94	France	1994	AF484953
B3	MVi/Caen.FRA/04	France	2004	DQ267505
B3	MVi/Lyon.FRA/20.04/1	France	2004	DQ267504
B3	MVi/GMB/93	Gambia	1993	AF484954
B3	MVi/GMB/96	Gambia	1996	AY059391
B3	MVi/Khartoum.SDN/33.97	Sudan	1997	AF453430
B3	MVi/Khartoum.SDN/36.97/2	Sudan	1997	AF453431
B3	MVi/Khartoum.SDN/28.00/5	Sudan	2000	AF453432
B3	MVi/Dande.BFA/17.01/1	Burkina Faso	2001	AY159463
B3	MVi/Padema.BFA/17.01/4	Burkina Faso	2001	AY159462
B3	MVi/Yaounde.CMR/01/9	Cameroon	2001	AF484952
B3	Mvi/Zliten.LBY/10.2007	Libya	2007	FN594772
B3	MVi/Zliten.LBY.14.09/4	Libya	2009	FN594724
B3	MVi/Sfax.TUN/18.02	Tunisia	2002	FN594773
B3	MVs/Gera.DEU/08.00	Germany	2000	AF480469

Genotype	Strain	Country	Collection year	Genbank accession No.
C1	MVi/Tokyo.JPN/84	Japan	1984	AY047365
C1	MVi/Osaka.JPN/84	Japan	1984	AB045296
C1	MVi/JPN/85/1	Japan	1985	Z80803
C1	MVi/Osaka.JPN/85	Japan	1985	AB045297
C1	MVi/Madrid.ESP/81	Spain	1981	Z80810
C1	MVs/Belfast.UNK/1/1955-sspe (UK88/55)	United Kingdom	1955	AF399851
C1	MVs/Belfast.UNK/2/1956-sspe (UK85/56)	United Kingdom	1956	AF399850
C2	MVi/Bethesda.Maryland.USA/77	United States	1977	M81898
C2	MVi/Illinois.USA/94	United States	1994	L46739
C2	MVi/Tennessee.USA/94	United States	1994	L46759
C2	MVi/Texas.USA/95	United States	1995	AF009599
C2	MVi/New Mexico.USA/32.96	United States	1996	AF009593
C2	MVi/Texas.USA/16.96	United States	1996	AF009594
C2	MVi/Utah.USA/23.96	United States	1996	AF009596
C2	MVi/Bilthoven.NLD/91/1	Netherlands	1991	L46747
C2	MVi/DEU/92/2	Germany	1992	Z80795
C2	MVi/DEU/92/3	Germany	1992	Z80799
C2	MVi/DEU/92/6	Germany	1992	Z80809
C2	MVi/DEU/92/7	Germany	1992	Z80808
C2	MVi/Tuebingen.DEU/24.00	Germany	2000	AF480468
C2	MVi/KALAA.MAR/10.99/1	Morocco	1999	AF458657
C2	MVi/Casablanca.MAR/19.03/2	Morocco	2003	DQ267500
C2	MVi/Taroudant.MAR/19.03/4	Morocco	2003	DQ267502
C2	MVi/Madrid.ESP/92/4	Spain	1992	Z80814
C2	MVi/Madrid.ESP/92/5	Spain	1992	Z80813
C2	MVi/Montreal.CAN/14.84	Canada	1984	AF410973
C2	MVi/Reuler.LUX/13.96	Luxembourg	1996	Y19086
C2	MVi/Reuler.LUX/14.96/1	Luxembourg	1996	Y19087
C2	MVi/Reuler.LUX/16.96/1	Luxembourg	1996	Y19089
C2	MVi/Reuler.LUX/16.96/2	Luxembourg	1996	Y19090
C2	MVi/Walferdange.LUX/15.96	Luxembourg	1996	Y19092
C2	MVi/Luxembourg.LUX/23.97	Luxembourg	1997	Y19097
C2	MVs/Toulon.FRA/08.07	France	2007	HM562898
D1	MVs/Belfast.GBR/60	United Kingdom	1960	AY077712
D1	MVs/Belfast.GBR/69	United Kingdom	1969	AY077713
D1	MVi/Bristol.GBR/74/1	United Kingdom	1974	Z80805
D1	MVi/Bristol.GBR/74/2	United Kingdom	1974	Z80804
D2	MVi/Johannesburg.ZAF/88/1	South Africa	1988	AF085198
D2	Mvi/Lusaka.ZMB/34.01	Zambia	2001	DQ845409
D2	MVi/Lusaka.ZMB/39.01	Zambia	2001	DQ845412
D2	MVi/Lusaka.ZMB/4.02	Zambia	2002	DQ845414
D3	MVi/Davis.California.USA/87	United States	1987	U97349
D3	MVi/Chicago.Illinois.USA/89	United States	1989	M81895
D3	MVi/Illinois.USA/89/2	United States	1989	L46736
D3	MVi/California.USA/90	United States	1990	L46725
D3	MVi/Guam.USA/94	United States	1994	L46734
D3	MVi/California.USA/32.96/	United States	1996	AF009581
D3	MVi/California.USA/8.04/	United States	2004	JN635409
D3	MVi/Goroka.PNG/26.99	Papua New Guinea	1999	AB075200
D3	MVi/Goroka.PNG/42.99/4	Papua New Guinea	1999	AB075201
D3	MVi/Taipeh.Taiwan.CHN/94	China	1994	AJ250060
D3	MVi/Hwalian.TWN/18.03/2	China	2003	EU914221
D3	MVi/Osaka.JPN/87/2	Japan	1987	AB045299
D3	MVi/Osaka C.JPN/14.98/1	Japan	1998	AF384225
D3	MVi/Tokyo.JPN/99	Japan	1999	AB089380
D3	MVi/Osaka C.JPN/22.00	Japan	2000	AF384233

Genotype	Strain	Country	Collection year	Genbank accession No.
D4	MVi/Amsterdam.NLD/3.98	Netherlands	1998	AJ250067
D4	MVi/Coast-Kwale.KEN/31.02/1	Kenya	2002	AY249265
D4	MVi/Nairobi.KEN/23.02	Kenya	2002	AY249261
D4	MVs/Central.KEN/24.02	Kenya	2002	AY249263
D4	MVs/Nyanza.KEN/24.02/1	Kenya	2002	AY249264
D4	MVi/ETH/99/1	Ethiopia	1999	AF280807
D4	MVi/ETH/99/2	Ethiopia	1999	AF280805
D4	MVi/Glasgow.GBR/14.94	United Kingdom	1994	GQ331933
D4	MVi/Karachi.PAK/89/1	Pakistan	1989	AB003182
D4	MVi/Karachi.PAK/89/2	Pakistan	1989	AB003179
D4	Mvi/Koungou.MAY.FRA/31.05/3	France	2005	EF428160
D4	MVi/Lyon.FRA/24.06/2	France	2006	EF428168
D4	Mvs/Reims.FRA/08.08	France	2008	GQ428195
D4	MVi/Montreal.CAN/38.89	Canada	1989	AF079554
D4	MVi/Washington.USA/95	United States	1995	AF009572
D4	MVi/Minnesota.USA/28.96	United States	1996	AF009571
D4	Mvi/Florida.USA/19.09	United States	2009	JN635403
D4	MVi/Phokhara.NPL/5.99	Nepal	1999	AJ250065
D4	MVs/Zagreb.HRV/48.03	Croatia	2003	AY594288
D4	MVs/Zagreb.CRO/30.06 (SSPE)	Croatia	2006	FJ475059
D4	MVs/Novosibirsk.RUS/49.00	Russia	2000	AY633620
D4	MVs/Vijaywada.Ind/51.06	India	2006	FJ387132
D4	MVs/Kangra.Ind/39.06/2	India	2006	FJ387138
D4	MVi/GULBARGA.IND/50.06	India	2006	FJ387146
D4	MVi/KOLAR.IND/49.06	India	2006	FJ387145
D4	MVs/Bijnore.Ind/08.06	India	2006	FJ387153
D4	MVs/Papumpare.Ind/51.07/2	India	2007	FJ387134
D4	MVs/Kargil.Ind/25.07/2	India	2007	FJ387139
D4	MVi/Perambalur.Ind/17.07	India	2007	FJ765087
D4	MVs/GANGTOK.IND/27.08/2	India	2008	FJ765072
D4	MVs/Minasgerais.BRA/41.11/	Brazil	2011	KC291548
D4	MVs/Riograndedosul.BRA/13.11/	Brazil	2011	KC291543
D5	MVi/Bangkok.THA/93	Thailand	1993	AF009580
D5	MVi/Bangkok.THA/94	Thailand	1994	AF009575
D5	MVi/Colorado.USA/94	United States	1994	L46727
D5	MVi/Nebraska.USA/94	United States	1994	L46745
D5	MVi/Washington.USA/94	United States	1994	L46766
D5	MVi/Massachusetts.USA/3.96	United States	1996	AF009577
D5	MVi/Washington.USA/19.96	United States	1996	AF009579
D5	MVi/Washington.USA/18.08/1	United States	2008	JN635405
D5	MVi/PLW/93	Palau	1993	L46757
D5	MVi/Osaka.JPN/91	Japan	1991	AB045300
D5	MVi/Osaka C.JPN/12.98	Japan	1998	AF384222
D5	MVi/Osaka C.JPN/32.98	Japan	1998	AF384235
D5	MVi/Osaka C.JPN/9.00/1	Japan	2000	AF384241
D5	MVi/Tokyo.JPN/00/1	Japan	2000	AB089379
D5	MVi/Toronto.CAN/20.96	Canada	1996	AF410977
D5	MVi/Tuebingen.DEU/10.08	Germany	2008	GQ121274
D5	MVs/SaintMande.FRA/09.08	France	2008	GQ428197

Genotype	Strain	Country	Collection year	Genbank accession No.
D6	MVi/New Jersey.USA/94	United States	1994	L46749
D6	MVi/Michigan.USA/95	United States	1995	AF009588
D6	MVi/Alaska.USA/6.96	United States	1996	AF009590
D6	MVi/Berlin.DEU/47.00	Germany	2000	AF480474
D6	MVi/Vancouver.CAN/7.97	Canada	1997	AF410978
D6	MVi/DNK/96/10	Denmark	1996	AF172974
D6	MVs/Glasgow.UNK/1990s-SSPE (UK125/90s)	United Kingdom	1990	AF504041
D6	MVi/GBR/94/4	United Kingdom	1994	Y16101
D6	MVi/Dudelange.LUX/25.96	Luxembourg	1996	Y19093
D6	MVi/Luxembourg.LUX/30.97/1	Luxembourg	1997	Y19098
D6	MVi/Luxembourg.LUX/30.97/2	Luxembourg	1997	Y19099
D6	MVi/Luxembourg.LUX/31.97	Luxembourg	1997	Y19100
D6	MVi/Luxembourg.LUX/24.01	Luxembourg	2001	FJ869876
D6	MVi/Madrid.ESP/94/7	Spain	1994	Z80823
D6	MVi/Madrid.ESP/94/3	Spain	1994	Z80816
D6	MVi/Madrid.ESP/95/2	Spain	1995	Z80825
D6	MVi/Madrid.ESP/96	Spain	1996	Z80832
D6	MVs/Novosibirsk.RUS/5.03	Russia	2003	AY523581
D6	MVi/Novokuzneck.RUS/44.03/1	Russia	2003	HM801955
D6	MVi/Moscow.RUS/3.04/	Russia	2004	HM801957
D6	MVi/NizhnyNovgorod.RUS/35.05/	Russia	2005	HM801937
D6	MVi/Rostov-na-Donu.RUS/15.05	Russia	2005	HM801935
D6	MVi/Orenburg.RUS/12.06	Russia	2006	HM801942
D6	MVs/Zagreb.CRO/47.02/[D6]_SSPE	Croatia	2002	DQ227318
D6	MVs/Zagreb.CRO/08.03/_SSPE	Croatia	2003	DQ227320
D6	MVi/Smorgon.BLR/20.06/1	Belarus	2006	HM801905
D7	MVi/Duesseldorf.DEU/10.00	Germany	2000	AF480471
D7	MVi/Greifswald.DEU/10.00/1	Germany	2000	AF480472
D7	MVi/Paris.FRA/01/1	France	2001	AY585728
D7	MVi/Lyon.FRA/03/1	France	2003	AY585725
D7	MV/Marseille.FRA/03/1	France	2003	AY585726
D7	MVi/Vic.AUS/16.85	Australia	1985	AF247202
D7	MVi/Vic.AUS/51.86	Australia	1986	AF247195
D7	MVi/Vic.AUS/29.88	Australia	1988	AF247198
D7	MVi/Vic.AUS/42.88	Australia	1988	AF247200
D7	MVi/Illinois.USA/50.99	United States	1999	AY043461
D7	MVi/California.USA/16.03	United States	2003	JN635410
D7	MVs/London.GBR/80	United Kingdom	1980	AF399848
D7	MVs/Nottingham2.UNK/1980s-SSPE (UK98/80s)	United Kingdom	1980	AF504040
D7	MVs/Nottingham1.UNK/1980s-SSPE (UK111/80s)	United Kingdom	1980	AY077714
D7	MVs/Bang.IND/5.03/SSPE	India	2003	DQ987235
D7	MVi/Chennai.IND/38.03	India	2003	DQ987234
D7	MVs/Pune.IND/11.04/1	India	2004	DQ987233
D7	MVs/Pune.Ind/10.07	India	2007	FJ387157
D8	MVi/BenniMellal.MAR/05/3	Morocco	2005	DQ779204
D8	MVi/Berlin.DEU/10.10/	Germany	2010	JQ417681
D8	MVi/Essen.DEU/12.10/	Germany	2010	JQ417684
D8	MVi/ETH/98	Ethiopia	1998	AF280806
D8	MVi/GBR/94/3	United Kingdom	1994	U29285
D8	MVi/Hetauda.NPL/2.99	Nepal	1999	AJ250063
D8	MVi/Janakpur.NPL/2.99/1	Nepal	1999	AJ250061
D8	MVi/Kathmandu.NPL/5.99	Nepal	1999	AJ250064
D8	Mvi/Sydney.AUS/2.03	Australia	2003	DQ852613
D8	MVi/Washington.USA/12.99	United States	1999	AY043462
D8	MVi/Texas.USA/4.07	United States	2007	JN635407
D8	MVi/Virginia.USA/15.09	United States	2009	JN635404
D8	MVs/UP.IND/42.01/10	India	2001	JN995537
D8	MVs/UP.IND/42.04/10	India	2004	JN995540
D8	MVs/Pune.Ind/48.05	India	2005	FJ387147
D8	MVi/BIJAPUR.IND/39.06	India	2006	FJ387142
D8	MVi/GADAG.IND/45.06	India	2006	FJ387144
D8	MVi/KOPPAL.IND/39.06	India	2006	FJ387141
D8	MVi/MYSORE.IND/44.06	India	2006	FJ387143
D8	MVi/Vellore.Ind/14.06	India	2006	FJ765086
D8	MVs/Bijnore.Ind/07.06/2	India	2006	FJ387154
D8	MVs/Chandigarh.IND/14.06	India	2006	FJ387136
D8	MVs/Mayurbhanj.Ind/42.06/2	India	2006	FJ387150
D8	MVs/PortBlair.Ind/06.06/1/OF	India	2006	FJ387130
D8	MVs/Pune.Ind/45.06/1	India	2006	FJ387148
D8	MVs/PURULIA.IND/38.06	India	2006	FJ387156
D8	MVs/Vijaywada.Ind/50.06	India	2006	FJ387133
D8	MVs/GANGTOK.IND/22.08	India	2008	FJ765071
D8	MVi/Tumkur.Ind/04.08/3	India	2008	FJ719488
D8	MVi/Vicenza.ITA/12.10/2	Italy	2010	KC117298
D8	MVi/Montreal.CAN/19.98	Canada	1998	AF410985
D8	MVs/Ontario.CAN/30.13/	Canada	2013	KF574437

Genotype	Strain	Country	Collection year	Genbank accession No.
D9	MVi/Yamagata.JPN/5.04	Japan	2004	AB186911
D9	MVi/Aichi.JPN/44.06	Japan	2006	AB426901
D9	MVi/Yamagata.JPN/12.09	Japan	2009	AB509377
D9	Mvi/Nice.FRA/20.08/2	France	2008	GQ428194
D9	MVs/NT.AUS/20.99	Australia	1999	AY127854
D9	MVi/Vic.AUS/12.99	Australia	1999	AY127853
D10	MVi/Kampala.UGA/51.00.1	Uganda	2000	AY923213
D10	MVi/Kampala.UGA/3.01	Uganda	2001	AY923214
D11	MVi/Menglian.Yunnan.CHN/47.09	China	2009	GU440576
E	MVi/DEU/71	Germany	1971	Z80797
E	MVi/Montreal.CAN/11.87	Canada	1987	AF410974
E	MVi/USA/70	United States	1970	Z80798
F	MVs/Madrid.SPA/94 SSPE	Spain	1994	Z80830
G1	MVi/Berkeley.California.USA/2.83	United States	1983	AF079553
G2	MVi/Amsterdam.NLD/49.97	Netherlands	1997	AF171231
G2	MVi/Jakarta.IDN/32.99	Indonesia	1999	AF243851
G3	MVi/Gresik.IDN/18.02	Indonesia	2002	AY184218
G3	MVs/Vic.AUS/24.99	Australia	1999	AF353621
H1	MVi/Amsterdam.NLD/27.97	Netherlands	1997	AJ250066
H1	MVi/GBR/96	United Kingdom	1996	Y16098
H1	MVi/Shandong.CHN/93/2	China	1993	AF045196
H1	MVi/Hunan.CHN/93/6	China	1993	AF045193
H1	MVi/Hunan.CHN/93/7	China	1993	AF045201
H1	MVi/Beijing.CHN/94/4	China	1994	AF045194
H1	MVi/Hebei.CHN/94/2	China	1994	AF045200
H1	MVi/Hunan.CHN/94/7	China	1994	AF045202
H1	MVi/Taoyuan.TWN.CHN/24.94/1	China	1994	EU914213
H1	MVi/CHN/97	China	1997	Y16096
H1	MVi/Shenyang.CHN/22.99	China	1999	GQ338160
H1	MVi/Taichung.TWN.CHN/40.02/2	China	2002	EU914219
H1	MVs/Taichung.TWN.CHN/36.02	China	2002	EU914218
H1	MVs/Taipei.TWN.CHN/26.02	China	2002	EU914216
H1	MVs/Taoyuan.TWN.CHN/45.01	China	2001	EU914215
H1	MVi/Zhejiang.CHN/02/2	China	2002	AY556538
H1	MVi/Taoyuan.TWN.CHN/20.03	China	2003	EU914222
H1	MVi/Ninbbo.Zhejiang.CHN/30.04/02	China	2004	DQ192531
H1	MVi/Ninbbo.Zhejiang.CHN/08.05/02	China	2005	DQ192532
H1	MVi/Zhejiang.CHN/10.05/2	China	2005	DQ011611
H1	MVi/Zhejiang.CHN/7.05/4	China	2005	DQ211902
H1	MVi/Zhejiang.CHN/9.05/10	China	2005	DQ011610
H1	MVs/Yunnan.CHN/19.06	China	2006	FJ161211
H1	MVi/Sofia.BGR/19.05	Bulgaria	2005	FJ808736
H1	MVi/Tokyo.JPN/00/2	Japan	2000	AB089378
H1	MVs/Kwangju.KOR/46.00	South Korea	2000	AY027629
H1	MVi/Pennsylvania.USA/20.09	United States	2009	JN635411
H2	MVi/Beijing.CHN/94/1	China	1994	AF045203
H2	MVi/Hanoi.VNM/23.98/1	Viet Nam	1998	AY026867
H2	MVi/Hoabinh.VNM/12.98/4	Viet Nam	1998	AY026869

Table S2. Models compared by AICM.

Model		AICM (SE)
Clock model	Strict clock	45762.7 (±0.515)
	Uncorrelated lognormal relaxed clock	45771.8 (±2.808)
	**Uncorrelated exponential relaxed cl[illegible]**	**45389.8 (±0.837)**
	Random local clock	45883.5 (±1.834)
Demographic model	Constant size	45389.8 (±1.400)
	**Exponential growth**	**45377.6 (±0.937)**

Data and models with bold letters are used in this study.

AICM, Akaike's information criterion through MCMC; SE, Standerd error.
